# Concerns Regarding the Methodology of a Psychological Inoculation Meta-Analysis on Misinformation

**DOI:** 10.2196/64430

**Published:** 2025-08-28

**Authors:** Daniel Loughnan, Aart van Stekelenburg, J Loes Pouwels, Mariska Kleemans

**Affiliations:** 1 Behavioural Science Institute Radboud University Nijmegen The Netherlands

**Keywords:** psychological inoculation, misinformation, discernment, sharing

The study “Psychological Inoculation for Credibility Assessment, Sharing Intention, and Discernment of Misinformation: Systematic Review and Meta-Analysis” [1] was published in volume 25 of the *Journal of Medical Internet Research*. It is the only meta-analysis we know of that aims to synthesize effects pertaining to all inoculation interventions against misinformation. It has been cited widely, including within an American Psychological Association consensus statement [2]. The importance of the reliability of these findings to this domain of research cannot be overstated.

Following assessment of the work and communication with one of the authors, we have some serious concerns we wish to share.

## Incorrect Inclusion and Exclusion of Effects (by Dependent Variable)

### Misinformation Credibility Assessment

At least 11 (weighted 35.7%) studies should have been excluded: 8 pertained to constructs other than a misinformation credibility assessment, and 3 included results for composite scales only.

### Real Information Credibility Assessment

At least 11 (weighted 41.1%) studies should have been excluded as the effects pertained to different constructs. At least 1 additional reviewed study should have been included.

### Credibility Discernment

By the stated decision rules, at least 2 other studies appearing in both the misinformation and real information credibility assessment categories should have been included (N=1215).

### Misinformation Sharing Intention

At least 1 (weighted 8.2%) study should have been excluded as it compared two inoculation conditions that were not randomly assigned.

### Real Information Sharing Intention

At least 3 (weighted 19.3%) studies should have been excluded as they pertained to different constructs or did not include a no-intervention control.

## Wrong Experimental Conditions Included

Six sets of extracted data pertained to experimental groups that did not represent the effect of interest. This affected meta-analytic findings for credibility assessments of misinformation and real information, and intentions to share real information.

## Implausible Sample Size Estimates

The authors estimated the SE from the standardized effect sizes (*d*) and sample sizes by condition (n) [3]. Where only unstandardized effects were reported, n was also used to calculate *d.* As such, the *d* and their SEs were functions of n. Importantly, n, *d*, and SE constitute all the information used in the meta-analysis.

For 3 studies reporting unstandardized effects, authors made implausible, notably incorrect estimates of n. This affected findings for credibility assessments of misinformation and real information. Multiple other errors regarding n values were also noted.

## Incorrect Conceptualization: Active Versus Passive

Lu et al [1] include a moderation analysis by intervention type: active versus passive. In active inoculation, participants contribute to the refutation process [4,5], but Lu et al [1] erroneously classified interventions by medium rather than participant contributions to the refutation process. Incorrect classification occurred for at least 9 high-powered studies.

These represent the most important findings of our analysis, which was constrained to appraisals of the supplementary materials, the primary studies, and two email exchanges with a representative author. We request this letter’s publication to raise awareness of these concerns. We further ask that all issues detailed in the full report be adequately addressed.

## Appendix

Multimedia Appendix 1 Full report on Lu et al.

Multimedia Appendix 2 Supplement to the full report on Lu et al.

Multimedia Appendix 3 Primary studies included in Lu et al's meta-analysis.

Multimedia Appendix 4 Data spreadsheet for full report on Lu et al.

## Data Availability

The full report and supplementary documents are available in the Open Science Framework repository [6] and in Multimedia Appendices 1-4.
