# Supplementary material for: Concerns Regarding the Methodology of a Psychological Inoculation Meta-Analysis on Misinformation
Source: J Med Internet Res. 2025 Aug 28;27:e64430. doi: 10.2196/64430 (PMC12428163; doi:10.2196/64430)
Supplement: Multimedia Appendix 1 [file jmir_v27i1e64430_app1.pdf]

7 March 2025

Full Report and Supplement to a Summary Letter to the Editor of *JMIR*, Re:

Lu, C., Hu, B., Li, Q., Bi, C., & Ju, X.-D. (2023). Psychological inoculation for credibility assessment, sharing intention, and discernment of misinformation: Systematic review and meta-analysis. *Journal of Medical Internet Research*, *25,* e49255.

Lu et al. (2023) is a systematic review and meta-analysis intended to determine the effects of psychological inoculation interventions on susceptibility to misinformation. It focusses on two outcomes when engaging with information: (1) assessments of its credibility and (2) intentions to share it. Each of these outcomes is considered in the context of a) *misinformation*, defined in the review as 'information that is false, but not created with the intention of causing harm' (Lu et al., 2023, p. 2; Wardle & Derakhshan, 2017), b) *real information,* that being unproblematic non-misinformation, and c) *discernment*, which is the ability of individuals to discriminate between misinformation and real information. In incorporating these six outcome measures, the effects submitted to meta-analysis pertain to: misinformation credibility assessment, real information credibility assessment, credibility discernment, misinformation sharing intentions, real information sharing intentions, and sharing discernment. The work also accounts for several moderation analyses: intervention strategy (*content-based* versus '*technology-based'* inoculation), intervention type (*active* versus *passive*), theme (*climate change*, *politics*, *health*, or *other*), measurement time (*immediate posttest*, *up to one week later*, *two weeks or more later*), team (*van der Linden group* or *other*), and intervention design (i.e., testing protocol: *pre-test/posttest* or *posttest only*).

Following, various consequential issues affecting the quality of the work are outlined. They include: 1) the incorrect inclusion and exclusion of studies and effects in the meta-analysis, 2) the inclusion of data pertaining to experimental groups that do not represent the effects of interest, 3) implausible and notably incorrect estimates of sample sizes by condition that could meaningfully bias the meta-analytic findings, 4) the incorrect conceptualization of active versus passive inoculation interventions impacting a key moderation analysis, and 5) other concerns and irregularities.

To supplement this summary report is a spreadsheet which features data extracted from Lu et al.'s (2023) supplementary materials, and corresponding data we extracted from the same studies. There is also a supplementary document discussing topics such as contrasting experimental paradigms, constructs, and effects of interest as they pertain to the Lu et al. (2023) study. Additionally, as the reference list of included studies provided by Lu et al. (2023) in their supplementary materials details a different set of articles, an accurate list is provided. These materials are available via https://osf.io/yfmvh/. Other materials that may be of use are those provided by Lu et al. (2023), available via https://osf.io/v8pxq/.

This report is not the result of an exhaustive analysis of the entire systematic search and meta-analytic process. We have limited our enquiries to the studies that appear in the review, the data as presented in the provided data extraction spreadsheets, reference to R scripts where they are supplied, and two email exchanges with a representative author. Methods pertaining to moderator analyses have not been assessed apart from noting some consequential errors in the categorization of active and passive interventions. We have not sought to replicate the literature search, the

screening process, or statistical analyses, and we make no determinations regarding the quality of those or any other elements of the study beyond those addressed here.

Studies included in the review that are cited in this report, which in some cases are in fact different samples from the same study, are denoted in the same way Lu and colleagues did in their data extraction spreadsheets (e.g., study one of Cook et al., 2017 is given as Cook2017_1, and sample two of study two of Basol et al., 2021 is given as Basol2021_2). The term 'real information' to denote reliable or unproblematic non-misinformation is retained from Lu et al. (2023) for the sake of consistency.

1. Incorrect Inclusion and Exclusion of Studies and Effects

This section presents details regarding specific studies or effects that have been erroneously included or excluded in the Lu et al. (2023) meta-analysis. They are arranged by the dependent variable of interest.

a) Misinformation Credibility Assessment

The pooled effects in this category were from 31 studies from 20 papers. We assessed that at least 11 studies (Weight = 35.7%) should not have been included:

- Apuke2022, Ma2023_1, and Ma2023_2 used composite scales including items that represented both misinformation and real information, with the misinformation items reverse coded to provide a scale of discernment. This was considered by Lu and colleagues to not fit their conceptualization of credibility discernment, and since data were not publicly available to allow for reanalysis, these studies did not meet the decision rule for inclusion in the credibility discernment category (Hu Bo, personal communication, 23 April 2024). However, mean ratings of the composite scales were erroneously included as

misinformation credibility assessments where they were in fact assessments of the credibility of both misinformation and real information. Further, Apuke2022 did not randomly assign participants to conditions, so should have been excluded as per exclusion criteria. Additionally, this study compares the delivery method, video-based or class-based, of an inoculation intervention, not an intervention versus no-intervention control condition as per the inclusion criteria.

- Bowman2021a and Bowman2021b measured source credibility. Lu et al. (2023) took reports of the perceived credibility of an organization that attacked another with misinformation as assessments of the credibility of misinformation. Source credibility of the attacking organization was measured with items such as 'I perceive this organization to be trustworthy/untrustworthy'. The measure was thus an assessment of the perceived credibility of an organization after exposure to a misinforming message, not an assessment of the credibility of misinformation (see the supplementary document with notes on contrasting experimental paradigms, constructs, and effects of interest for a discussion on constructs other than information credibility assessments, such as source credibility).
- Piltch-Loeb2022 did not randomly assign participants to conditions, so should have been excluded from the review by the exclusion criteria. Further, they assessed whether participants recognized techniques such as the use of 'scary music' or 'weird colors', not how they assessed the credibility of misinformation. However, Piltch-Loeb2022 note that they did present questions regarding the credibility of misinformation that was presented in an attack message, but

responses on this variable were not included in their results. According to the Lu et al. (2023) supplementary materials, the raw data were not reanalyzed so these responses could not have been included.

- Cook2017_1 measured multiple dependent variables after exposure to climate misinformation. Judging by the effect size data extracted, Lu et al. (2023) took trust in contrarian scientists as a measure of misinformation credibility assessment. Items on this 5-item scale included: 'I do not trust the things that scientists challenging the consensus view of climate change say about climate science'. This operationalizes trust in some species of scientist after exposure to misinformation, not an assessment of the credibility of misinformation.
- Roozenbeek2022_7 assessed participants' ability to recognize a rhetorical technique of persuasion via a single-response, 4-choice multiple-choice test. The recognition of a technique does not inform if the information was seen as credible or not and does not constitute a credibility rating (see the relevant supplementary document for more discussion).
- Zerback2021_1 and Zerback2021_2 assessed opinion change and opinion certainty regarding current affairs relating to Russia and Syria after exposure to news teasers with astroturfing comments (presenting an orchestrated campaign as initiated by members of the public). These were not assessments of the credibility of misinformation but instead measures of participants' attitudes on current affairs.
- Maertens2020, a replication of van der Linden2017, took perceived scientific consensus as their dependent variable. This construct represents participants' perceptions of the consensus in the climate science community regarding the

occurrence of anthropogenetic climate change, from 0-100%. This measure did not directly assess how credible the misinforming attack was perceived to be, but rather, how willing participants were to update initial perceptions after exposure to information (an accurate consensus statement and a misinforming attack message). Outcomes will have in part depended on factors other than assessments of information credibility (see the supplementary document for more discussion on this dependent variable as a proxy for information credibility assessment).

b) Real Information Credibility Assessment

The pooled effects in this category were from 26 studies from 18 papers. We assessed that at least 11 studies (Weight = 41.1%) should not have been included, and that one study that appears elsewhere in the review (*N* = 198) should have been:

- van der Linden2017 and Williams2020, a replication of van der Linden2017, took perceived scientific consensus as their dependent variable. This construct represents participants' perceptions of the consensus in the climate science community regarding the occurrence of anthropogenetic climate change, from 0-100%. This measure did not directly assess how credible the misinforming attack was perceived to be. Outcomes will have in part depended on factors other than assessments of information credibility (see notes on Maertens2020 above and the relevant supplementary document for more discussion).
- Bowman2021a and Bowman2021b measured source credibility. Lu et al. (2023) took reports of the perceived credibility of an organization that was attacked by another with misinformation and responded with a refutation as assessments of the credibility of real information. Source credibility assessments were

measured with items such as ‘I perceive this organization to be trustworthy/untrustworthy’. The measure was an assessment of the perceived credibility of an organization, not credibility of real information.

- Cook 2017_1, Cook2017_2 and Schmid-Petri2022, a replication of Cook2017_2, each considered multiple dependent variables after exposure to climate misinformation. Judging by the effect size data extracted from each study, for Cook2017_1, Lu et al. (2023) took trust in climate scientists as a measure of real information credibility assessment, while for Cook2017_2 and Schmid-Petri2022 they took acceptance of anthropogenic climate change. In neither case does the dependent variable represent real information credibility assessment. It is unclear why different dependent variables were preferred for extraction between the studies as each included both.
- Iles2021 considered multiple dependent variables after exposure to an anti-science misinforming message. Comparing effect sizes between the meta-analysis and original work, the data extracted by Lu et al. (2023) appear to pertain to the outcome measure ‘an intention to follow official mammography recommendations’. This behavioral intention was thus taken as a measure of real information credibility assessment, though it is not. Further, Iles2021 included a measure of ‘perceived argument strength’ of a misinforming message, measured with items including ‘The article I read is believable’. Results pertaining to this item would have allowed this study to be included in the misinformation credibility assessment category. Data were available by request.
- Dai2022, Jiang2022, and Park2022 measured attitudes and intentions regarding a vaccine after exposure to an attack message. (Dai2022 also included

alternative smoking, e.g., vaping). Attitudes were measured on the dimensions bad/good, harmful/beneficial, foolish/wise, threatening/assuring, and risky/safe. Such a measure does not represent credibility assessments of information. Further, for Park2022 there was no appropriate control group as participants in the comparator condition received a supportive message that the inoculation condition did not. This is a common comparison in traditional inoculation studies, often alongside a no-intervention control, but it is a comparison between interventions to reduce persuasion by an attack message and thus different to a control group comparison to assess the protective effect of an inoculation intervention compared to no intervention.

- Basol2020 included three real news test items. They were not featured in the published paper, but data are available and were included in a reanalysis cited in Lu et al. (2023; Modirrousta-Galian & Higham, 2023). Basol2020 was thus eligible for inclusion in the real information credibility assessment category by Lu et al.'s (2023) decision rule (Hu Bo, personal communication, 23 April 2024).

c) Credibility Discernment

The pooled effects in this category were from 12 studies from five papers. We assessed that at least two studies (*N* = 1215) that appear elsewhere in the review should have been included:

- Agley2021 included four misinformation test items and three real information test items. These were rated on 'believability', pre- and post-intervention. Responses are thus misinformation and real information credibility assessments, respectively, and the difference between them a valid measure of credibility discernment. Data are available online and were accessed and

apparently reanalyzed by Lu et al. (2023). However, from supplementary materials it appears a reanalysis was conducted only with respect to misinformation credibility assessment, but not real information credibility assessment or credibility discernment (R scripts are not supplied and what the reanalysis was is unclear). By the inclusion and exclusion criteria and the authors' decision rule (Hu Bo, personal communication, 23 April 2024), effects should have been included in the credibility discernment category. Further, based on previous decisions made by Lu et al. (2023), it appears that they may have extracted data pertaining to trust in science, that being a dependent variable similar to trust in climate scientists that was taken from Cook2017_1, as a real information credibility assessment. If they did use trust in science, effects should not have been included in any credibility assessment category.

- Basol2020 included three real news test items. They were not featured in the published paper, but data are available and were included in a reanalysis cited in Lu et al. (2023; Modirrousta-Galian & Higham, 2023). Basol2020 was thus eligible for inclusion in the real information credibility assessment category by Lu et al.'s (2023) decision rule (Hu Bo, personal communication, 23 April 2024), as well as the misinformation credibility assessment category, and thus eligible for inclusion in the credibility discernment category.

d) Misinformation Sharing Intentions

The pooled effects in this category were from 12 studies from six papers. We assessed that at least one study (Weight = 8.2%) should not have been included:

- Apuke2022 did not randomly assign participants to conditions, so should have been excluded. Additionally, this study compares the delivery method, video-

based or class-based, of a single inoculation intervention, not intervention and control conditions.

e) Real Information Sharing Intentions

The pooled effects in this category were from 11 studies from four papers. We assessed that at least three studies (Weight = 19.3%) should not have been included:

- Brinson2022a and Brinson2022b measured counterarguing intent towards pro-vaccine content using a 7-point bipolar scale (-3 to 3). Participants were asked: 'If that message you just saw appeared on your social media feed, you would… (-3) Immediately post a comment about what's wrong with it, (-2) Indicate my disagreement with a negative emoticon, (-1) Do nothing, but tell friends I saw "fake news" about the COVID-19 vaccine, (0) Scroll past and forget about it, (1) Do nothing, but tell friends I saw some good news about the COVID-19 vaccine, (2) Indicate my agreement with a positive emoticon, (3) Immediately share it with my social network'. Only the last of the seven options mentions sharing. Lu et al. (2023) did not access the raw data to make an analysis of sharing (3) versus not sharing (-3 to 2), rather, they included counterarguing intent in relation to pro-vaccine statements as real information sharing intentions, which it is not.
- Park2022 did not include an appropriate control group as participants in the comparator condition received a supportive message. A supportive message condition is not a control condition that allows for no-intervention comparisons. Further, this study measured word of mouth intentions in relation to information about vaccines. Items included "I would encourage family members or relatives to get HPV vaccines" and "I would encourage friends to get HPV vaccines". It is

debatable whether this constitutes sharing as commonly understood in this field of research (i.e., of posts on social media).

2. Inclusions of Wrong Experimental Conditions

This section details six instances in which data attributable to the wrong experimental conditions were extracted for inclusion:

- van der Linden2017 and replications Williams2020 and Maertens2020: Judging by the extracted samples sizes by condition for all studies, and by the effects extracted from van der Linden2017 and Maertens2020, none of the data from these studies reflects the effects of interest.
    - For van der Linden2017 and Williams 2020, the 'consensus treatment' group was taken as the inoculation condition. But this group was only provided a statement on scientific consensus (i.e., '97% of climate scientists have concluded that human caused climate change is happening'). Consequentially, for those two studies, no inoculation group is represented by the data included in the meta-analysis.
    - The data extracted from Maertens2020 does include that pertaining to the inoculation condition, but the wrong control group was included. Control group participants in these studies were not presented with any information, but only asked their perception of the scientific consensus on anthropogenetic climate change. This condition (no consensus information, no misinformation) represents the population baseline perception and cannot be meaningfully compared to the inoculation condition (consensus information, inoculation, misinformation) to infer a

protective effect against misinformation from inoculation. The relevant condition to include would have been the 'balance' group, who, like the inoculation group, were exposed to a consensus statement followed by misinformation attacking it. In that instance, the inoculation group would have differed from the comparator group only on the inclusion of an inoculation intervention before the misinforming attack, thereby allowing for inferences of a protective effect. The same error was made in extracting data from van der Linden2017.

  - It is not possible to infer from sample sizes by condition which control group was taken for Williams2020 and it is not clear which group comparison the extracted effect size pertains to, but the comparator condition was either the no-information control group (consistent with the other studies), the counter-message group (attack message only), or the inoculation condition. None of those are the appropriate comparator group for assessing an effect of inoculation against misinformation.

- Park2022: There was no appropriate control group in this study as participants in the comparator condition received a supportive message. A supportive message condition is not a control that allows for no-intervention comparisons. Instead, it allows for comparisons between interventions to reduce susceptibility to misinformation. This affected the meta-analytic findings for real information credibility assessment and sharing intention.
- Schmid-Petri2022: Judging by the extracted sample sizes by condition, Lu et al. (2023) included the 'misinformation only' group as the control, and 'inoculation only' as treatment. There was, however, an 'inoculation preceding

misinformation' condition. The consequence of this is that the included inoculation group was not exposed to misinformation, but the control group was. Lu and colleagues did not do this for Cook2017_2 though, which Schmid-Petri2022 replicates. This affected the meta-analytic finding for real information credibility assessment.

- Green2022: The intervention and control group data appear the wrong way around in the data extraction spreadsheet pertaining to real information credibility assessment, but not misinformation credibility assessment.

Note: In several instances where there are multiple inoculation conditions representing different styles of inoculation, topics, or other differences, it is unclear which treatment condition or conditions were included. Decision rules such as these were not detailed.

3. Implausible Sample Size Estimates Affecting Meta-Analytic Findings

Lu et al. (2023) report they extracted the means, standard deviations, and samples sizes for each study. However, on relatively many occasions samples sizes by condition for relevant experimental groups were not in fact obtainable. On those occasions, the research team made estimates (Hu Bo, personal communication, 15 April 2024). Some of these estimates were implausible and notably different to best estimates, with implications for meta-analytic findings.

To reduce bias that could have been introduced by taking variances reported by differing methods (Hu Bo, personal communication, 15 April 2024), all standard errors (*SE*) were estimated from the standardized effect sizes (*d*) and sample sizes by condition (*n*; Hedges, 1981). Further, R scripts show that where *d* was not reported it was calculated from the raw effect sizes, *sd*, and *n*. The implication of the above is that

many of the inputted standardized effect sizes along with all *SE* informing the weight attributed to each study in the meta-analysis, were functions of *n*. This makes the accuracy of any estimations of *n* particularly important.

- Amazeen2022 reports $N = 540$, randomly assigned to seven conditions. It was a 3 (inoculation message type: none, generic, specific) x 2 (debunk message: yes/no) design with an offset control (no misinforming attack message). The relevant conditions for comparison would include the 'inoculation message type' conditions that did not receive a debunking message ('none' as the control, then one or both the 'generic' and 'specific' inoculation conditions as the treatment). Those conditions constitute two or three of the seven (which treatment condition or conditions were included is unclear). The posttest was immediate and there was no attrition, so the best estimate for the control condition sample size is n_C = 540/7 = 77. If one inoculation condition had been chosen as the treatment then the best estimate for the intervention condition would be $n_I = 77$, or if effects were combined, $n_I = 154$. Lu et al. (2023) reported the total relevant sample size as $N = 386$, with $n = 193$ in each condition. They apparently included five of the seven conditions in the relevant sample, then took *N/*2 to determine the estimates for each *n*. Uniformly dividing the total relevant *N* by two was a method employed in estimating sample sizes by condition described by Hu Bo (personal communication, 23 April 2024). Of note is that Amazeen2022 did not report standardized effects, so the incorrectly estimated *n*s were used to also estimate *d*, confounding the meta-analytic result for misinformation credibility assessment.

- Dai2022 gives the total samples size as *N* = 543 randomly assigned to seven conditions: 2 (topic) x 3 (inoculation conditions) with an offset control (no inoculation). All conditions were exposed to an attack message. The posttest was immediate and there was no attrition, so the best estimate for the control condition would be n_C = 543/7 = 78. It is unclear which conditions were included. Lu et al. (2023) reported the relevant sample size as *N* = 286, with *n* = 143 in each condition. It is unclear how these numbers, 286 in the context of 543 participants randomly assigned to seven conditions, relate to the sample and method. Dai2022 did not report standardized effects, so here too the incorrectly estimated *n* was used to calculate not just *SE* but also *d*, confounding the result for real information credibility assessment.
- Vraga2019 gives the total samples size as *N* = 406 randomly assigned to three conditions, one of which was the control condition. The posttest was immediate and there was no attrition. The best estimate for the control condition is thus n_C = 406/3 = 135. The remaining two conditions were inoculation conditions (different types). It is unclear which conditions were included. Lu et al. (2023) reported the relevant sample size as *N* = 406, with *n* = 203 in each condition. Vraga2019 did not report standardized effects, so the incorrectly estimated *n* was used to also calculate *d*, confounding the results for real information credibility assessment.
- 11 other instances of incorrect sample sizes were noted. At least some appear to be imputation errors rather than those arising from a methodological issue. These are relatively small discrepancies in comparison to those above (see the supplementary spreadsheet for details). However, considering the potential impact on meta-analytic findings arising from Lu et al.'s (2023) methods of

estimation, and considering also that seven of those errors would have had effects for a single meta-analytic finding, that of misinformation credibility assessment, these additional discrepancies cannot be assumed negligible.

4. Incorrect Conceptualization of Active versus Passive Interventions

Active psychological inoculation interventions are those in which participants actively participate in producing counterarguments within the preemptive refutational process of psychological inoculation (Banas & Rains, 2010; Compton & Pfau, 2005; McGuire, 1961, 1964). This distinction may be illustrated by the active and passive inoculation interventions tested by Green2022: the passive condition involved reading about the use of media techniques which may mislead, while the active condition was the same but with the addition of a task creating tweets that feature the techniques. Importantly, the medium does not dictate the classification, but the way participants engage with the refutation does. Inconsistent with the definition of active and passive inoculation interventions, and even active versus passive experiential learning, Lu et al. (2023) used intervention medium to inform the categorization of interventions; they considered videos and games to be inherently active and all other interventions, such as text-based interventions and in-class courses, to be inherently passive. This misunderstanding led to the misclassification of at least nine high-powered studies. The following issues pertaining to the active versus passive moderation analysis are noted:

- The videos featured in Roozenbeek2022_1-7 and Lewandowsky2021 are erroneously classified as active inoculation. They are not, as subjects that watch the videos have no active involvement in producing refutations. These non-interactive videos would not be considered examples of active learning either,

and we are aware of no studies that refer to them as active interventions of any sort.

- The data extracted from Green2022 was categorized as passive but pertained to the active text-based intervention included in that study (which was then accidentally inverted for real information assessment, as detailed in section 2).
- The video interventions tested by Piltch-Loeb2022 are erroneously categorized as graphic interventions, but, presumably due to this error, correctly labelled as passive inoculation in the data extraction spreadsheets.

Note: We have not gone through every intervention to check whether they are correctly classified as active or passive. However, we have determined that the classification criteria are misunderstood by Lu et al. (2023) and it is thus likely there are more errors. The errors noted here, though, are enough to invalidate the results considering the high power of Roozenbeek2022_1-7 and Lewandowsky2021, especially Roozenbeek2022_7 that alone accounts for over half of the total sample size of the meta-analysis.

5. Other Concerns and Irregulates

Uninterpreted Adjustments for Publication Bias

After adjustments using the Duval and Tweedie trim-and-fill method, the findings for five of the six meta-analytic effect size estimates were rendered statistically non-significant. The finding for the remaining meta-analytic estimate for credibility discernment was not rendered insignificant after adjustments, although the funnel plot and PET-PEESE test both suggested the presence of publication bias. The possibility of this body of research suffering from publication bias, and the non-significant effects

after statistical adjustments, were ignored in the discussion, conclusion, limitations, and abstract.

Generalizing from Few Publications and Interventions

The credibility discernment and sharing discernment meta-analytic estimates, findings which Lu et al. (2023) note will be of most interest to researchers in the field, included effects from studies that appeared in just five and two papers, respectively. In both cases, Roozenbeek2022_1-6 are included, studies which account for approximately 63% and 82% of the weight in each category. For such weight to be given to studies from one set of related experiments testing related interventions (short, animated videos) that differ markedly to most other interventions, has obvious implications for the generalizability of findings to all inoculation interventions. Yet findings are ubiquitously interpreted as pertaining to inoculation generally. Another note regarding Roozenbeek2022 is that Roozenbeek2022_7 accounted for only 3.6% of the weighting of the meta-analytic effect for misinformation credibility assessment despite having a sample size of N = 22,632, over 20x that of the next largest total sample size. It is unclear why its notably lower variance does not translate to higher weighting.

'Technology-based'

Throughout the paper Lu et al. (2023) refer to 'technology-based inoculation' that they claim, 'focuses on the technology behind the information' (p. 2). Inoculation interventions to address misinformation susceptibility do not inoculate against information characterized by their use of a technology, such as information mediated by a computer, a television, or a newspaper. Rather, they focus on the application of rhetorical *techniques* of persuasion (e.g., ad hominem attacks or presenting fake

experts; Cook et al., 2017). The term to be substituted for 'technology-based' in Lu et al. (2023) is 'technique-based' (also sometimes termed 'logic-based').

Definition of Misinformation

Relatively many primary studies included do not adhere to the narrow definition of misinformation offered by Lu and colleagues, that being information that is 'false, but not created with the intention of causing harm' (Lu et al., 2023, p. 2; Wardle & Derakhshan, 2017). This definition excludes false information created with the intention to harm (i.e., disinformation) or true-but-misleading (i.e., 'or otherwise misleading') information. Where definitions differ, so to do test stimuli and items, and the conceptualization of susceptibility to misinformation.

Description of Tests

Lu et al. (2023) state that when testing in this field of study, 'researchers present a series of social media posts or news statements containing true or false information to subjects (Pennycook et al., 2020; Roozenbeek & van der Linden, 2019), after which a binary or Likert scale is used to allow the subjects to assess the information's credibility and their willingness to share it' (Lu et al., 2023, p. 2). While this accurately describes some tests concerned with assessments of information credibility and intentions to share it, it does not describe any of the studies concerned with attitudes and opinions regarding things other than information credibility, or the explicit identification of rhetorical techniques (e.g., via a multichoice question). It should be noted too that in most of the included studies that do indeed consider information credibility assessments, misinformation test items are not verified as false, but rather, include the use of a fallacious rhetorical technique of persuasion. Though either conceptualization may pertain to information credibility assessments, a test of the ability to accurately

assess the credibility (or reliability or trustworthiness) of a claim based on its use of a fallacious rhetorical technique is not the same as a test of the ability to accurately assess the credibility of a claim based on its underlying truth or falsity independent of its use of such techniques.

Conclusion

We believe the evidence presented in this report is sufficient to cast serious doubt on the reliability of Lu et al.'s (2023) meta-analytic findings. This is consequential as this is the only meta-analysis we are aware of that aims to synthesize all effects pertaining to the reduction of susceptibility to misinformation attributable to psychological inoculation interventions generally. As such, it has already been cited in a substantial number of works in the field, including within an APA consensus statement on health misinformation (van der Linden et al., 2023). This thus requires action from *JMIR* to inform readers of the concerns presented here. Further, we call for these issues to be adequately addressed by Lu et al. (2023). Until such time, we recommend researchers refrain from citing Lu et al. (2023) as credible meta-analytic evidence.

References

Banas, J. A., & Rains, S. A. (2010). A meta-analysis of research on inoculation theory. *Communication Monographs*, *77*(3), 281–311. https://doi.org/10.1080/03637751003758193

Basol, M., Roozenbeek, J., & van der Linden, S. (2020). Good news about Bad News: Gamified inoculation boosts confidence and cognitive immunity against fake news. *Journal of Cognition*, *3*(1), 1-9. https://doi.org/10.5334/joc.91

Compton, J. A., & Pfau, M. (2005). Inoculation theory of resistance to influence at maturity: Recent progress in theory development and application and suggestions for future research. *Annals of the International Communication Association*, *29*(1), 97–146. https://doi.org/10.1080/23808985.2005.11679045

Cook, J., Lewandowsky, S., & Ecker, U. K. H. (2017). Neutralizing misinformation through inoculation: Exposing misleading argumentation techniques reduces their influence. *PLoS ONE, 12*(5), e0175799. https://doi.org/10.1371/journal.pone.0175799

Hedges, L. V. (1981). Distribution theory for Glass's estimator of effect size and related estimators. *Journal of Educational and Behavioral Statistics*. https://doi.org/10.3102/10769986006002107

Lu, C., Hu, B., Li, Q., Bi, C., & Ju, X.-D. (2023). Psychological inoculation for credibility assessment, sharing intention, and discernment of misinformation: Systematic review and meta-analysis. *Journal of Medical Internet Research*, *25,* e49255.

McGuire, W. J. (1964). Some contemporary approaches. In *Advances in Experimental Social Psychology* (Vol. 1, pp. 191–229). Elsevier. https://doi.org/10.1016/S0065-2601(08)60052-0

McGuire, W. J., & Papageorgis, D. (1961). The relative efficacy of various types of prior belief-defense in producing immunity against persuasion. *The Journal of Abnormal and Social Psychology*, *62*(2), 327-337.

Modirrousta-Galian A. & Higham P.A. (2023). Gamified inoculation interventions do not improve discrimination between true and fake news: Reanalyzing existing research with receiver operating characteristic analysis. *Journal of Experimental Psychology. General*, *152*(9), 2411–2437. https://doi.org/10.1037/xge0001395

Pennycook, G., McPhetres, J., Zhang, Y., Lu, J. G., & Rand, D. G. (2020). Fighting COVID-19 misinformation on social media: Experimental evidence for a scalable accuracy-nudge intervention. *Psychological Science*, *31*(7), 770-780.

Roozenbeek, J., & van der Linden, S. (2019). Fake news game confers psychological resistance against online misinformation. *Palgrave Communications*, *5*(65). https://doi.org/10.1057/s41599-019-0279-9

van der Linden, S., Albarracín, D., Fazio, L. K., Freelon, D., Roozenbeek, J., Swire-Thompson, B., & Van Bavel, J. J. (2023). Using psychological science to understand and fight health misinformation: An APA consensus statement. *American Psychological Association*.

Wardle, C., & Derakhshan, H. (2017). *Information disorder: Toward an interdisciplinary framework for research and policymaking* (Vol. 27, pp. 1-107). Strasbourg: Council of Europe.
