# Supplementary material for: Concerns Regarding the Methodology of a Psychological Inoculation Meta-Analysis on Misinformation
Source: J Med Internet Res. 2025 Aug 28;27:e64430. doi: 10.2196/64430 (PMC12428163; doi:10.2196/64430)
Supplement: Multimedia Appendix 2 [file jmir_v27i1e64430_app2.pdf]

Supplement to the Full Report on Lu et al. (2023): Notes on Experimental Paradigms, Constructs, and Effects of Interest

Lu et al. (2023) describe in their introduction a version of a contemporary paradigm for testing psychological inoculation against misinformation as representative of studies in the field: 'researchers present a series of social media posts or news statements containing true or false information to subjects (Pennycook et al., 2020; Roozenbeek & van der Linden, 2019a), after which a binary or Likert scale is used to allow the subjects to assess the information's credibility and their willingness to share it' (Lu et al., 2023, p. 2). However, studies concerned with changes in attitudes and which thus met the inclusion criterion '[s]tudies not related to credibility assessment' were apparently retained regardless (Lu et al., 2023, p. 5). The review goes on to make no distinction between different testing paradigms, constructs, and effects of interest reflected in the included primary studies. This appears to be a major factor contributing to the inappropriate inclusion of effects in meta-analytic categories concerned with information credibility assessments.

Two Experimental Paradigms

In the traditional, fact-based inoculation paradigm (Banas & Rains, 2010; McGuire, 1964), a two-sided preemptive refutation, that being a weak argument followed by a strong rebuttal, is delivered ahead of a persuasive attack message intended to shift participant attitudes. An effect of inoculation is demonstrated via post-exposure resistance to attitude change relative to a control group, and/or a one-sided supportive message condition. In the contemporary, technique-based inoculation against misinformation paradigm (Basol et al., 2020), a preemptive refutation is delivered ahead of the presentation of two classes of short, text-based test stimuli that

simulate misinformation and real information (e.g., unreliable or reliable social media posts, respectively). An effect of inoculation is demonstrated via the accuracy of the credibility assessments of those items relative to a control group. The traditional and contemporary paradigms are thus concerned with different outcomes: attitude protection from persuasive arguments versus the accurate assessment of test stimuli that simulate misinformation and real information. These are not interchangeable dependent variables, and only one can be considered a credibility assessment.

Contrasting Constructs and Effects of Interest

On multiple occasions the meta-analytic work synthesized effects pertaining to different constructs. Often, this was a result of attitudes toward things other than the credibility of some information being taken as information credibility assessments. This was in each case inappropriate. To illustrate, subjects may assess some information presented to be credible or not, but that may not affect other attitudes, beliefs, or opinions. For example, assessments of source credibility for an organization after exposure to some communication, as featured in Bowman22021a and Bowman2021b, could hinge greatly upon previous perceptions of that organization's credibility. In such a case, perceived source credibility may be unaffected by how credible or otherwise a communication was assessed to be. In these two studies, perception of source credibility for one organization that attacked another with misinformation were taken as misinformation credibility assessments, and source credibility for the attacked organization that refuted the attack message were taken as real information credibility assessments. However, neither were credibility assessments of information. Only assessments of information credibility are information credibility assessments that may be appropriately synthesized toward a single meta-analytic finding for that construct.

Another study in which measures of attitudes within the same study were taken to represent misinformation and real information credibility assessments was Cook2017_1. This study is included in the misinformation credibility assessment category, taking trust in climate-change-contrarian scientists as the dependent variable. It was also included in the real information credibility assessment category, taking trust in climate scientists as the dependent variable. In this case also, test items employed in the traditional experimental paradigm were erroneously treated as if they were stimuli representing misinformation and real information to be rated on credibility.

The inconsistent treatment of effects from related studies concerned with changes in attitudes shows that the distinctions between these constructs were sometimes not clear to the researchers. For example, van der Linden2017 and Williams2020 are categorized as addressing real information credibility assessment and Maertens2020 as addressing misinformation credibility assessment, although the latter studies are replications of van der Linden2017 and each study includes the same test materials and dependent variable. Relatedly, Cook2017_1, Cook2017_2, and Schmid-Petri2022 that replicates Cook2017_2, all include the dependent variables trust in climate scientists and acceptance of anthropogenic climate change. For Cook2017_1 trust in climate scientists was taken as real information credibility assessment, but for the other two studies acceptance of anthropogenic climate change was.

Other effects that did not pertain to the featured construct and were thus improperly included for meta-analysis include measures of credibility discernment from composite scales that were treated as misinformation credibility assessments (Apuke2022, Ma2023_1, and Ma2023_2), and counterarguing intent that was treated as real information sharing intent (Brinson2022a and Brinson2022b).

Recognition of Rhetorical Techniques via a Multiple-Choice Test

Roozenbeek2022_7 assessed participants' ability to recognize a rhetorical technique of persuasion via a single-response, 4-choice multiple-choice test. The recognition of a technique does not inform if the information was seen as credible or not. For example, recognition that an appeal for charitable donations heavily features the use of emotive language, one of the study's target rhetorical techniques, does not assume the information communicated was not considered credible.

Perceptions of Scientific Consensus

Three studies, van der Linden2017, Maertens2020, and Williams2020, included the dependent variable perceived scientific consensus for anthropogenic climate change. This construct concerns an estimate of the percentage of climate scientists that have concluded that anthropogenic climate change is occurring. It could be argued that if participants are told the consensus is 97% and one group updates their posttest estimate toward 97% more than the other, we might reasonably infer that group found the consensus information more credible. While we acknowledge that perceived scientific consensus after the provision of a consensus statement would likely be a better proxy for information credibility assessment than, say, trust in climate scientists, we do not consider this is automatically the case.

A measure of perceived scientific consensus does not directly assess how credible some information is assessed to be. Instead, it assesses a perception that is perhaps affected by such information. While there is strong evidence that consensus information can have a substantial effect on perceived scientific consensus, this does not mean this outcome variable is a meaningful indicator of information credibility. There might be a range of factors beyond the perceived credibility of the target

information that would explain an effect of a consensus message on perceived consensus (e.g., political orientation, trust in scientists). Thus, perceived scientific consensus does not reliably operationalize information credibility assessment and synthesizing these effects with credibility assessments can be expected to confound meta-analytic findings.

References

Banas, J. A., & Rains, S. A. (2010). A meta-analysis of research on inoculation theory. *Communication Monographs*, *77*(3), 281–311. https://doi.org/10.1080/03637751003758193

Basol, M., Roozenbeek, J., & van der Linden, S. (2020). Good news about Bad News: Gamified inoculation boosts confidence and cognitive immunity against fake news. *Journal of Cognition*, *3*(1), 1-9. https://doi.org/10.5334/joc.91

Lu, C., Hu, B., Li, Q., Bi, C., & Ju, X.-D. (2023). Psychological inoculation for credibility assessment, sharing intention, and discernment of misinformation: Systematic review and meta-analysis. *Journal of Medical Internet Research*, *25,* e49255.

McGuire, W. J. (1964). Some contemporary approaches. In *Advances in Experimental Social Psychology* (Vol. 1, pp. 191–229). Elsevier. https://doi.org/10.1016/S0065-2601(08)60052-0
