# Supplementary material for: Concerns Regarding the Methodology of a Psychological Inoculation Meta-Analysis on Misinformation
Source: J Med Internet Res. 2025 Aug 28;27:e64430. doi: 10.2196/64430 (PMC12428163; doi:10.2196/64430)
Supplement: Multimedia Appendix 3 [file jmir_v27i1e64430_app3.pdf]

Primary Studies Included in the Lu et al. (2023) Meta-analysis

Agley, J., Xiao, Y., Thompson, E. E., Chen, X., & Golzarri-Arroyo, L. (2021). Intervening on trust in science to reduce belief in COVID-19 misinformation and increase COVID-19 preventive behavioral intentions: Randomized controlled trial. *Journal of Medical Internet Research*, *23*(10), e32425. https://doi.org/10.2196/32425

Amazeen, M. A., Krishna, A., & Eschmann, R. (2022). Cutting the bunk: Comparing the solo and aggregate effects of prebunking and debunking Covid-19 vaccine misinformation. *Science Communication*, *44*(4), 387–417. https://doi.org/10.1177/10755470221111558

Apuke, O. D., Omar, B., Tunca, E. A., & Gever, C. V. (2023). The effect of visual multimedia instructions against fake news spread: A quasi-experimental study with Nigerian students. *Journal of Librarianship and Information Science*, *55*(3), 694–703. https://doi.org/10.1177/09610006221096477

Basol, M., Roozenbeek, J., Berriche, M., Uenal, F., McClanahan, W. P., & van der Linden, S. (2021). Towards psychological herd immunity: Cross-cultural evidence for two prebunking interventions against COVID-19 misinformation. *Big Data & Society*, *8*(1), 11-18. https://doi.org/10.1177/20539517211013868

Basol, M., Roozenbeek, J., & van der Linden, S. (2020). Good news about Bad News: Gamified inoculation boosts confidence and cognitive immunity against fake news. *Journal of Cognition*, *3*(1), 1-9. https://doi.org/10.5334/joc.91

Biddlestone, M., Roozenbeek, J., & van der Linden, S. (2023). Once (but not twice) upon a time: Narrative inoculation against conjunction errors indirectly reduces conspiracy beliefs and improves truth discernment. *Applied Cognitive Psychology*, *37*(2), 304–318. https://doi.org/10.1002/acp.4025

Boman, C. D. (2021). Examining characteristics of prebunking strategies to overcome PR disinformation attacks. *Public Relations Review*, *47*(5), 102105.

Boman, C. D., & Schneider, E. J. (2021). Finding an antidote: Testing the use of proactive crisis strategies to protect organizations from astroturf attacks. *Public Relations Review*, *47*(1), 102004. https://doi.org/10.1016/j.pubrev.2020.102004

Brinson, N. H. (2022). Resistance to persuasion: Examining the influence of political ideology on COVID-19 vaccine uptake hesitancy. *Frontiers in Communication*, *6*, 760847. https://doi.org/10.3389/fcomm.2021.760847

Cook, J., Lewandowsky, S., & Ecker, U. K. H. (2017). Neutralizing misinformation through inoculation: Exposing misleading argumentation techniques reduces their influence. *PLoS ONE, 12*(5), e0175799. https://doi.org/10.1371/journal.pone.0175799

Dai, Y., Jia, W., Fu, L., Sun, M., & Jiang, L. C. (2022). The effects of self-generated and other-generated eWOM in inoculating against misinformation. *Telematics and Informatics*, *71*, 101835. https://doi.org/10.1016/j.tele.2022.101835

Green, M., McShane, C. J., & Swinbourne, A. (2022). Active versus passive: Evaluating the effectiveness of inoculation techniques in relation to misinformation about climate change. *Australian Journal of Psychology*, *74*(1), 2113340. https://doi.org/10.1080/00049530.2022.2113340

Guan, T., Liu, T., & Yuan, R. (2021). Facing disinformation: Five methods to counter conspiracy theories amid the Covid-19 pandemic. *Comunicar*, *29*(69), 71–83. https://doi.org/10.3916/C69-2021-06

Iles, I. A., Gillman, A. S., Platter, H. N., Ferrer, R. A., & Klein, W. M. P. (2021). Investigating the potential of inoculation messages and self-affirmation in reducing the effects of health misinformation. *Science Communication*, *43*(6), 768–804. https://doi.org/10.1177/10755470211048480

Jiang, L. C., Sun, M., Chu, T. H., & Chia, S. C. (2022). Inoculation works and health advocacy backfires: Building resistance to COVID-19 vaccine misinformation in a low political trust context. *Frontiers in Psychology*, *13,* 976091 https://doi.org/10.3389/fpsyg.2022.976091

Lewandowsky, S., & Yesilada, M. (2021). Inoculating against the spread of Islamophobic and radical-Islamist disinformation. *Cognitive Research: Principles and Implications*, *6*(57). https://doi.org/10.1186/s41235-021-00323-z

Ma, J., Chen, Y., Zhu, H., & Gan, Y. (2023). Fighting COVID-19 misinformation through an online game based on the inoculation theory: Analyzing the mediating effects of perceived threat and persuasion knowledge. *International Journal of Environmental Research and Public Health*, *20,* 980. https://doi.org/10.3390/ijerph20020980

Maertens, R., Anseel, F., & van der Linden, S. (2020). Combatting climate change misinformation: Evidence for longevity of inoculation and consensus messaging effects. *Journal of Environmental Psychology, 70,* 101455. https://doi.org/10.1016/j.jenvp.2020.101455

Maertens, R., Roozenbeek, J., Basol, M., & van der Linden, S. (2021). Long-term effectiveness of inoculation against misinformation: Three longitudinal experiments. *Journal of Experimental Psychology: Applied*, *27*(1), 1–16. https://doi.org/10.1037/xap0000315

Park, E., Kim, S., & Cameron, G. T. (2022). Immunize the HPV vaccine rumors: Effects of inoculation messages and tone of voice on parental intention to vaccinate their children. *Journal of Community Health*, *47*(5), 790–799. https://doi.org/10.1007/s10900-022-01100-9

Piltch-Loeb, R., Su, M., Hughes, B., Testa, M., Goldberg, B., Braddock, K., Miller-Idriss, C., Maturo, V., & Savoia, E. (2022). Testing the efficacy of attitudinal inoculation videos to enhance COVID-19 vaccine acceptance: Quasi-experimental intervention trial. *JMIR Public Health and Surveillance*, *8*(6), e34615. https://doi.org/10.2196/34615

Roozenbeek, J., Maertens, R., McClanahan, W., & van der Linden, S. (2021). Disentangling item and testing effects in inoculation research on online misinformation: Solomon revisited. *Educational and Psychological Measurement*, *81*(2), 340–362. https://doi.org/10.1177/0013164420940378

Roozenbeek, J., & van der Linden, S. (2019). The fake news game: Actively inoculating against the risk of misinformation. *Journal of Risk Research*, *22*(5), 570–580. https://doi.org/10.1080/13669877.2018.1443491

Roozenbeek, J., van der Linden, S., Goldberg, B., Rathje, S., & Lewandowsky, S. (2022). Psychological inoculation improves resilience against misinformation on social media. *Science Advances*, *8*(34), eabo6254. https://doi.org/10.1126/sciadv.abo6254

Schmid-Petri, H., & Bürger, M. (2022). The effect of misinformation and inoculation: Replication of an experiment on the effect of false experts in the context of climate change communication. *Public Understanding of Science*, *31*(2), 152–167. https://doi.org/10.1177/09636625211024550

van der Linden, S., Leiserowitz, A., Rosenthal, S., & Maibach, E. (2017). Inoculating the public against misinformation about climate change. *Global Challenges*, *1*, 1600008. https://doi.org/10.1002/gch2.201600008

Vraga, E. K., Kim, S. C., & Cook, J. (2019). Testing logic-based and humor-based corrections for science, health, and political misinformation on social media. *Journal of Broadcasting & Electronic Media*, *63*(3), 393–414. https://doi.org/10.1080/08838151.2019.1653102

Williams, M. N., & Bond, C. M. C. (2020). A preregistered replication of “Inoculating the public against misinformation about climate change”. *Journal of Environmental Psychology*, *70*, 101456. https://doi.org/10.1016/j.jenvp.2020.101456

Zerback, T., Töpfl, F., & Knöpfle, M. (2021). The disconcerting potential of online disinformation: Persuasive effects of astroturfing comments and three strategies for inoculation against them. *New Media & Society*, *23*(5), 1080–1098. https://doi.org/10.1177/1461444820908530
